# Amino Acid Plasma Profiles from a Prolonged-Release Protein Substitute for Phenylketonuria: A Randomized, Single-Dose, Four-Way Crossover Trial in Healthy Volunteers

**DOI:** 10.3390/nu12061653

**Published:** 2020-06-02

**Authors:** Mika Scheinin, Anna Barassi, Jouni Junnila, Zsófia Lovró, Giorgio Reiner, Essi Sarkkinen, Anita MacDonald

**Affiliations:** 1 CRST Oy, Itäinen Pitkäkatu 4B, FI-20520 Turku, Finland; zsofia.lovro@crst.fi; 2 Institute of Biomedicine, University of Turku and TYKSLAB, Turku University Hospital, Kiinamyllynkatu 4-8, FI-20520 Turku, Finland; 3 APR Applied Pharma Research sa via Corti 5, CH-6828 Balerna, Switzerland; barassi.anna@libero.it (A.B.); giorgio.reiner@apr.ch (G.R.); 4 Oy 4Pharma Ltd., Arkadiankatu 7, FI-00100 Helsinki, Finland; jouni.junnila@4pharma.com; 5 Food and Nutrition, Oy Medfiles Ltd. (CRO), P. O. Box 1450, FI-70701 Kuopio, Finland; essi.sarkkinen@medfiles.eu; 6 Dietetic Department, Birmingham Women’s and Children’s Hospital NHS Foundation Trust, Birmingham B4 6NH, UK; Anita.Macdonald@nhs.net

**Keywords:** disorders of amino acid metabolism, phenylketonuria, dietary management, amino acid absorption, bioequivalence, prolonged release

## Abstract

Several disorders of amino acid (AA) metabolism are treated with a protein-restricted diet supplemented with specific AA mixtures. Delivery kinetics impacts AA absorption and plasma concentration profiles. We assessed plasma profiles after ingestion of an AA mixture engineered to prolong AA absorption with Physiomimic Technology^TM^ (Test) in a randomized, single-dose, four-way crossover trial in healthy volunteers (Trial Registration: ISRCTN11016729). In a two-step hypothesis, the primary endpoints were (i) significant reduction in peak plasma concentrations (C_max_) of essential amino acids (EAAs) while (ii) maintaining EAA bioavailability (AUC_0-300 min_) compared to a free AA mixture (Reference). Secondary endpoints included effects on plasma profiles of other AA groups and effects on several metabolic markers. Thirty subjects completed the study. Both co-primary endpoints were met: C_max_ for EAAs was 27% lower with the Test product compared to the Reference product (ratio, 0.726, *p* < 0.0001); overall plasma EAA levels from the two AA mixtures was within the pre-specified bioequivalence range (AUC_0-300min_ ratio, 0.890 (95% CI: 0.865, 0.915)). These findings were supported by the results of secondary endpoints. Prolongation of AA absorption was associated with modulation of several metabolic markers. It will be important to understand whether this can improve the long-term management of disorders of AA metabolism.

## 1. Introduction

The estimated global incidence of all inherited disorders of amino acid (AA) metabolism is 15/100,000 live births. Of these, phenylketonuria (PKU) represents the most common and significant disease entity, with an estimated global incidence of 7/100,000 [1], although birth incidence varies by ethnicity and consanguinity, and estimates are influenced by the level of ascertainment [2]. Many inherited disorders of AA metabolism require prompt initiation of treatment at birth and lifelong adherence to a protein-restricted diet supplemented with defined AA mixtures that lack specific AAs [3,4,5,6,7,8]. Important products downstream of a metabolic block may need to be supplemented, such as tyrosine in PKU, phenylalanine in tyrosinemia and cysteine in some forms of homocystinuria. The diet must be engineered to provide adequate vitamins, minerals and other nutrients normally obtained from natural protein-containing foods. These are usually provided through appropriate supplementation of the AA mixture. In all, more than 70 disorders of AA metabolism are managed with AA mixtures [9].

The plasma AA profile after ingesting free AAs is different from that of AAs administered as intact proteins: plasma levels peak faster and higher but decrease more quickly with free AAs [10]. The rate of absorption influences the fate of the ingested AAs. Fast absorption is associated with more AA oxidation, higher levels of blood urea nitrogen (BUN), and less protein accretion compared to slowly digested proteins [11,12,13]. This results in lower biological value for free AA mixtures compared to intact proteins [14]. In an effort to compensate for these differences, treatment guidelines for PKU recommend daily AA intake of up to 150% of the amounts recommended for healthy subjects, distributed through the day [15,16], with the objective of providing sufficient nitrogen assimilation and minimising protein catabolism.

The absorption kinetics of free AA mixtures used for the dietary treatment of disorders of AA metabolism have not been extensively investigated. Modifying the “immediate-release” absorption kinetics typically observed with free AAs to a more gradual kinetic profile with a less prominent peak may improve the efficiency of protein metabolism, reduce AA oxidation and the increase in BUN that occurs when large amounts of free AAs are ingested. Prolonging the uptake of AAs may also reduce the incidence and severity of catabolic episodes, thereby reducing fluctuations in blood phenylalanine levels [17].

Key kinetic parameters for investigating the effect of replacing “immediate-release” free AAs with prolonged-release AAs in the diet of such patients include the peak blood concentration (C_max_), which should be lower, and the overall extent of absorption expressed as the area under the concentration/time curve (AUC), which should be equivalent (Supplementary Figure S1). Prolonged release is also typically reflected by higher blood concentrations at later time points after intake (C_last_).

Physiomimic Technology^TM^ (PT^TM^) is a pharmaceutical process that results in small granules coated with functional additives—ethylcellulose and sodium alginate—that allow gradual release of their contents in the small intestine. PT^TM^ modifies the release and absorption of AAs, while masking their taste and odour, with positive effects on the typically unpleasant aftertaste of traditional AA formulations. This study was based on preliminary evidence obtained from a porcine model, where application of PT^TM^ to free AA mixtures reduced C_max_ by 18% while maintaining a similar overall increase in plasma AAs [18].

We conducted the present study in healthy adult volunteers to determine the effects on plasma AA profiles of a prolonged-release AA mixture formulated with PT^TM^, comparing it with an immediate-release formulation of the same AA mixture, a commercially available free AA mixture and a natural intact protein, casein. We also measured blood urea nitrogen (BUN), insulin and glucose levels to explore whether the AA release kinetics modulate these metabolic pathways.

## 2. Materials and Methods

### 2.1. Subjects

Enrolled subjects were healthy non-smoking men or women between the ages of 18 and 45 years with body weight between 55 and 85 kg and body mass index (BMI) ≤ 30 kg/m^2^; pregnant and breast-feeding women were excluded; subjects were excluded if they had health conditions that investigators considered might interfere with the study aims including, but not limited to gastrointestinal, renal or hepatic diseases, diabetes or other metabolic conditions. Full enrolment criteria are available in Supplementary Table S1.

At the screening visit, subjects underwent a physical examination, 12-lead ECG, comprehensive laboratory blood tests and received nutritional counselling for diet standardization. All study participants provided written informed consent. The study was conducted at CRST Oy in Turku, Finland, and was approved by the Ethics Committee of the Hospital District of Southwest Finland, Turku, Finland; Ref: 78/1801/2017. Trial Registration: ISRCTN11016729.

### 2.2. Study Design

This randomized, single-blind, four-way crossover trial tested the effects of a novel prolonged-release coating technology on the plasma profiles and bioavailability of AAs from a protein substitute for PKU. The primary comparison was between (i) the Test product, a prolonged-release AA formulation engineered with PT^TM^ containing vitamins, minerals and other nutrients, and (ii) the Reference product, a mixture containing identical proportions and quantities of free AAs, vitamins, minerals, other nutrients and functional additives (Supplementary Table S2). Sample size was calculated on both primary endpoints to detect a 20% change in C_max_ and bioequivalent overall increase in plasma EAAs with 90% power.

Secondary comparisons were made with (i) a commercially available AA mixture (MP) with a composition comparable to the Test and Reference products, and (ii) an intact slowly digested protein derived from cow’s milk (food-grade acid casein; 80 MESH; A.C.E.F., Italy). These comparisons are considered secondary because MP and casein have AA compositions that are quantitatively different from the Test and Reference products; casein differs also qualitatively from the other products, in that it lacks added micronutrients and minerals.

Subjects were assigned to one of 3 body weight categories: 60 kg (55–65.4), 70 kg (65.5–75.4), 80 kg (75.5–85). For 2 days before each test day, subjects were provided a standardized diet with daily energy and protein content corresponding to their body weight (60 kg: 1988 kcal/56.6 g protein, 70 kg: 2292 kcal/65.8 g protein, 80 kg: 2784 kcal/77.6 g protein). On the evening before each test day, subjects consumed a light meal with a standardised, low protein content (0.24 g/kg).

On each test day, after overnight fasting (10–12 h), subjects were assigned to receive one of the study products based on the randomisation sequence (Supplementary Figure S2).

**Figure 1:**
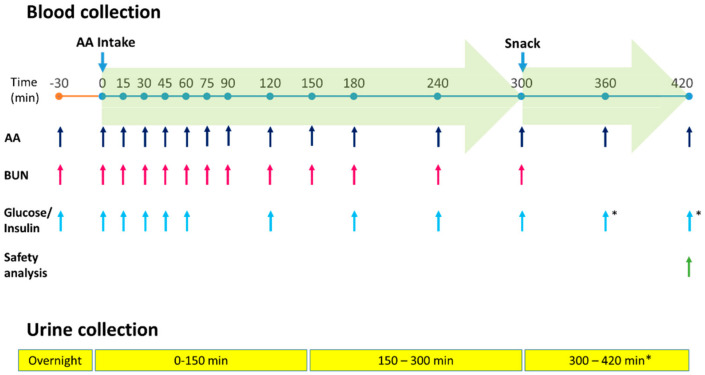
Sample collection and analysis schedule. AA, amino acids; BUN, blood urea nitrogen; *for safety assessment only.

A nominal dose of 0.4 g AA/kg, estimated to provide one third of the daily protein equivalent (PE) requirement generally prescribed to adults with PKU, was administered as a suspension in 300 mL of water. Dosing was adjusted by body weight: the 55–65.4 kg group receiving 24 g AAs (20 g PE), the 65.5–75.4 kg group receiving 28 g AAs (23.3 g PE) and the 75.5–85 kg group receiving 32 g AAs (26.6 g PE). A 300-kcal light snack containing mainly carbohydrates was provided 5 h after intake of the study products to prevent protein catabolism and consequent release of AAs into the bloodstream. The schedule of each test day is illustrated in Figure 1.

Fifteen timed venous blood samples were collected via an indwelling catheter up to 7 h after product intake. Concentrations of AAs, BUN, insulin and glucose were determined. Baseline values were derived from samples collected 30 min and just before dosing (0 min). Urine was collected on the morning of the test day (12 h over-night urine), and subsequent fractions were collected between 0 and 150 min and between 150 and 300 min to measure urea and other laboratory parameters; a fourth sample was collected between 300 and 420 min for safety laboratory assessments.

Test days were planned to be separated by washout periods of 9 to 14 d. The total study duration for each subject was planned to be approximately 38–70 d (screening period, 4 test days separated by 3 washout periods and a follow-up visit, if required).

### 2.3. Primary Endpoints

Two important aspects of prolonged release are the reduction of peak blood AA levels (C_max_) together with maintenance of the overall extent of absorption of an ingested dose, also expressed as bioavailability. Therefore, two co-primary endpoints were evaluated to compare the Test product vs. the Reference product:

1) Superiority in terms of the C_max_ in plasma essential amino acids (EAAs) and
2) Bioequivalence in terms of plasma AUC of the EAAs over the first 5 h after product intake (AUC_0-300min_); the AUC cut-off was set at 5 h to avoid potential changes in plasma AAs after the snack was administered.

The criteria for evaluating the primary endpoints were established a priori, with analysis of the second being contingent on meeting the first: (i) significantly lower C_max_ for EAAs with the Test product vs. Reference product; (ii) an equivalent EAA bioavailability over the first 5 h defined as a log-transformed AUC_0-300min_ ratio with a 90% CI in the range 0.80–1.25, based on guidelines for bioequivalence studies with drug products [19,20].

EAAs included tyrosine and arginine; tyrosine was included because it is conditionally essential in PKU and arginine because it is necessary for adequate growth in patients on diet regimens that are low in natural proteins.

### 2.4. Secondary Endpoints

Secondary endpoints addressed the plasma profiles for up to 7 h of EAAs and additional groups of AAs, including single AAs, large neutral AAs (LNAAs), branched-chain AAs (BCAAs) and total AAs. Secondary endpoints evaluated to compare the Test product vs. the Reference product were:

1) C_max_ of these AA groups in plasma;
2) Their bioequivalence in terms of plasma AUC over the first 7 hours after product intake (AUC_0-420 min_);
3) Their plasma concentration at the last measured time point (C_last_).

C_max_ and the AUC_0–420min_ ratio were compared as above.

### 2.5. Secondary Comparisons

Formal comparisons between the Test product and the MP comparator or casein were not performed due to differences in their AA compositions; however, we did make secondary comparisons between their plasma profiles, as described for the secondary endpoints.

We compared trends in blood levels of phenylalanine and tyrosine among the 3 phenylalanine-free AA preparations, because these AAs are critical in the treatment of patients with PKU. We also explored possible differences in blood insulin, glucose, BUN and urea excretion in urine.

Overall acceptability/palatability and subjective satiety were assessed 2 h after study products were ingested using questionnaires adapted from Flint et al. [21] and translated into Finnish.

### 2.6. Safety Assessments

On the first 3 test days, blood parameters for liver and kidney function were measured (aspartate aminotransferase, alanine aminotransferase, ɣ-glutamyl transferase, bilirubin, alkaline phosphatase, creatinine). Additionally, concentrations of albumin and prealbumin in serum were evaluated at the end of each of the first three test days. After the fourth test day, the examinations and tests used at screening were repeated. On all test days, urine parameters (creatinine, total protein, urate, calcium, phosphate, pH, urea) were evaluated. Subjects maintained a diary that was given to them on each test day and collected at the next visit. Adverse events were recorded. Tolerability of all study products was evaluated.

### 2.7. Statistical Analysis

Sample size calculation was based on both co-primary endpoints (C_max_ and AUC_0-300min_ of EAAs). With 90% power, 24 evaluable subjects were needed to demonstrate a ≥20% decrease in C_max_ and bioequivalence of AUC_0-300 min_ for EAAs comparing the Test product with the Reference product. Assuming a drop-out rate of 25%, this corresponded to randomising 32 subjects.

AUC was calculated using the trapezoidal rule. Equivalent or better bioavailability of the Test vs. Reference product corresponded to a log-transformed AUC ratio with a 90% CI of ≥0.80. Primary endpoints were analysed on log-transformed data. A linear mixed effect model appropriate for a four-period crossover design was fitted for the parameters. The statistical model included sequence, period and treatment as fixed effects, and “subject within sequence” and residual error term as random effects. The *p*-value was used for superiority testing for C_max_; in addition, a 90% CI for AUC_0-300min_ was calculated from the model for equivalence testing. CI estimates were converted by anti-log transformation to obtain ratios of geometric least square means. Comparable statistical methodology was used for the secondary parameters. All results are described as mean, standard deviation, geometric mean, median, and coefficient of variation. Comparisons of kinetic parameters between the Test product and both commercially available comparator products, MP and casein, and between the Reference product and MP and casein were calculated from the statistical models described above. Effects on the metabolic markers BUN, urine urea, insulin and glucose were compared with repeated measures of covariance (ANCOVA). Responses to the questionnaire on overall acceptability of the products were compared with Wilcoxon exact test.

## 3. Results

Of the 35 healthy volunteers enrolled, 30 completed the study according to the protocol: 15 women and 15 men, mean ± SD age 27.4 ± 8.2 years (range 18–43 years), mean ± SD body weight 70.0 ± 8.5 kg. Four subjects discontinued without receiving any dose of any product, and one subject was excluded from the per-protocol analysis because of a major protocol violation. This subject’s results were included in the safety evaluation.

### 3.1. Kinetic Outcomes

#### 3.1.1. Primary Endpoints

**Figure 2:**
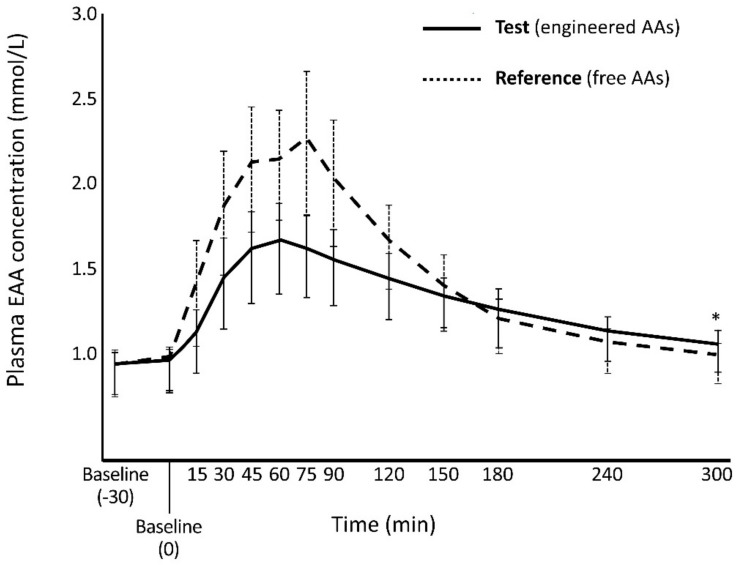
Plasma EAA concentrations after ingesting Test and Reference products. * Mean (SD) concentration after 5 h (C_last_) was significantly higher with the Test product, 1.06 (0.124) vs. 0.996 (0.117) mmol/L; *p* = 0.0012. ANCOVA test.

Both co-primary endpoints were met (Figure 2; Table 1). The C_max_ for EAAs was 27% lower with the Test product compared to the Reference product (ratio 0.726, *p* < 0.0001) whereas the overall increase in plasma EAAs (AUC_0-300_) from the two AA mixtures was within the pre-specified bioequivalence range (ratio, 0.890 (90% CI: 0.865, 0.915)).

#### 3.1.2. Secondary Endpoints

These findings were supported by the results of secondary endpoints on the plasma profiles of different classes of AAs. The C_max_ values for EAAs, LNAAs, BCAAs and total AAs were consistently significantly lower after intake of the Test product compared to the Reference product (Table 2), while the overall increase in plasma levels (AUC_0-420_) was in the bioequivalence range over the entire 7 h of observation (Table 3).

C_last_ values 7 h after intake of the Test product were significantly higher compared to C_last_ after the Reference product for all classes of AAs but not for total AAs (Table 4).

#### 3.1.3. Secondary Comparisons

Comparisons between the Test product and the comparator products MP and casein were considered secondary due to differences in their compositions. The Test product consistently showed significantly lower C_max_ values compared to both MP and casein (Table 5).

When compared to MP, the Test product demonstrated bioequivalent increases in plasma levels of EAAs, LNAAs and total AAs at 420 min (Table 6).

We evaluated effects on plasma tyrosine levels because, although all marketed AA mixtures for PKU contain high levels of tyrosine, it is not highly soluble and its bioavailability represents a critical point. Analyses showed that the Test and Reference products were equivalent (AUC_0-420_ ratio 1.052 (90% CI: 0.984, 1.125)), meaning that PT^TM^ did not alter the bioavailability of this AA, which is conditionally essential in PKU patients. Comparisons of plasma tyrosine profiles with MP and casein are not informative because of their different AA compositions compared to the Test and Reference products.

**Figure 3:**
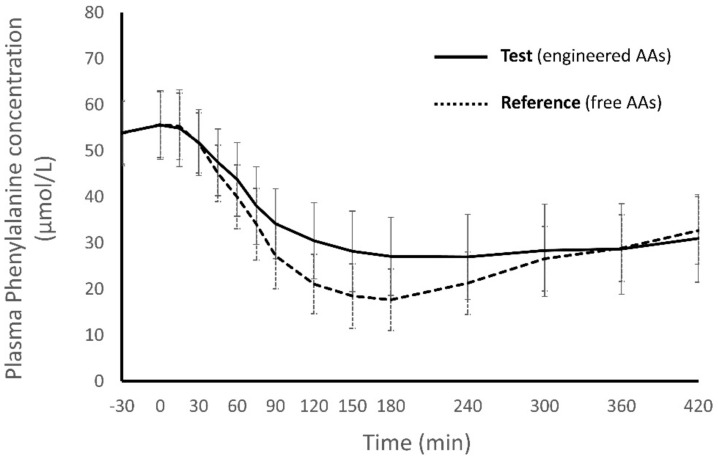
Plasma phenylalanine concentrations after intake of Test and Reference products. There was less variation in phenylalanine concentration after Test (*p* = 0.0046, ANCOVA).

Although phenylalanine was not present in the Test and Reference products, their effects on the kinetic profile of plasma phenylalanine were compared to determine if administering a prolonged release AA mixture influences this important parameter. Plasma phenylalanine fluctuations were less prominent after intake of the Test product vs. the Reference product (*p* = 0.0046, ANCOVA; Figure 3).

### 3.2. Markers of Metabolism

**Figure 4:**
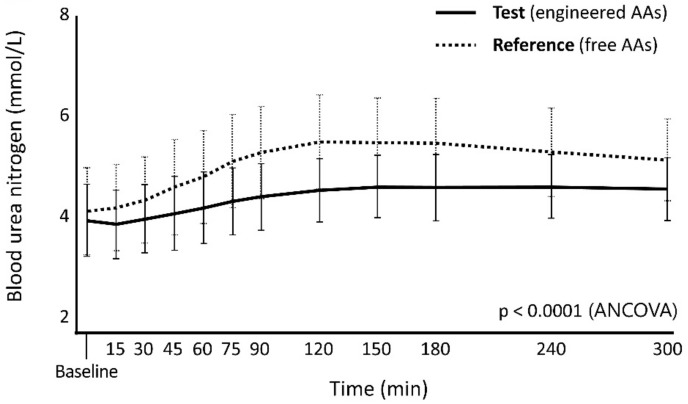
Blood urea nitrogen (BUN) measured at the indicated times after ingestion of Test and Reference products as an indicator of nitrogen utilization.

We measured changes in BUN and urine urea, two indicators of AA catabolism. A statistically significant difference emerged between the Test and the Reference products for both BUN (ANCOVA test: *p* < 0.0001; Figure 4) and urine urea excretion (ANCOVA test: *p* = 0.0074; Table 7).

This was supported by a finding of significantly lower mean (SD) quantity of urea excreted with the Test product vs. Reference (Table 7).

**Figure 5:**
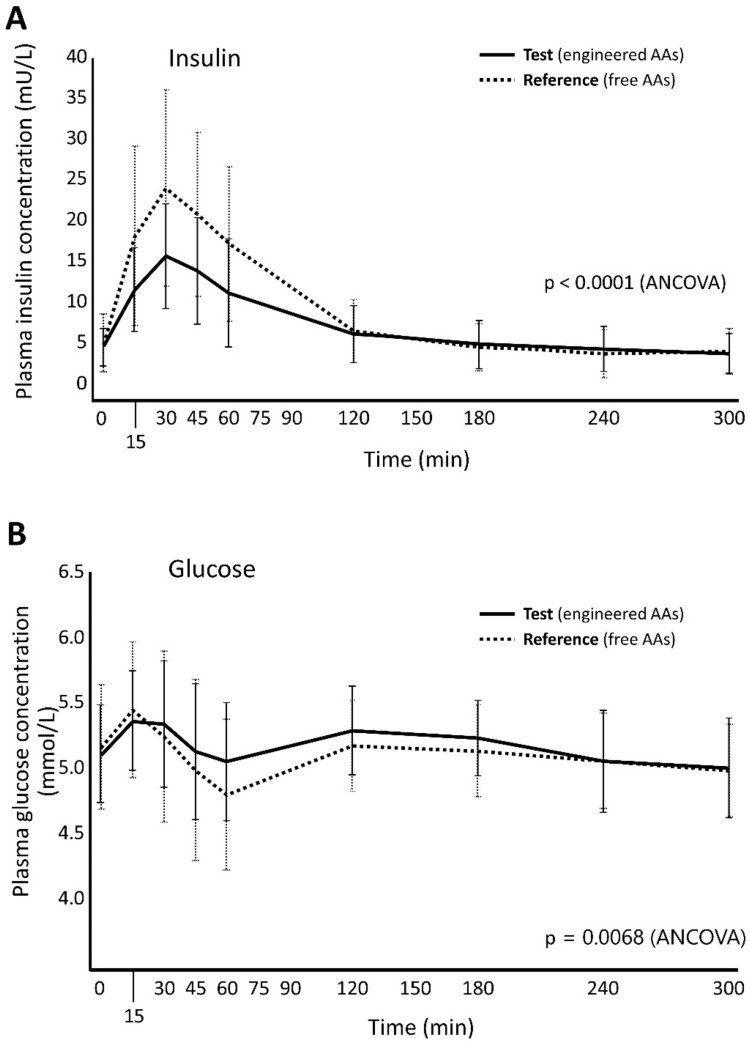
Plasma levels of (**A**) insulin and (**B**) glucose measured at the indicated times after ingestion of Test and Reference products.

We measured plasma levels of insulin and glucose to explore whether administering a prolonged-release AA mixture might influence these biomarkers of metabolism (Table 8). Analysing insulin curves over time for the products and constructing an RM-ANCOVA model revealed a statistically significant difference between the Test and the Reference products (Figure 5A; ANCOVA test: *p* < 0.0001). The curves for glucose mirror the curves for insulin: after insulin release from pancreatic beta cells, blood levels of glucose start to decrease as glucose is taken into muscle, fat and liver cells. The lower the insulin peak, the smaller the decrease of glucose levels.

After intake of the Test product, glucose levels did not decrease as much and were more constant over time compared to levels after intake of the Reference product. By analysing glucose curves over time for the products and constructing an RM-ANCOVA model, a statistically significant difference emerged between the Test and the Reference products (Figure 5B; ANCOVA test: *p* = 0.0068).

Regarding the subjects’ assessment of the products, there were no significant differences between products for subjectively evaluated palatability or sense of satiety, except for a higher rating on the item “overall liking” for casein compared to the Test product (*p* < 0.0006; Wilcoxon exact test).

### 3.3. Safety Profile

In the safety population (n = 31), 27 subjects (87%) reported at least one adverse event (AE). Most AEs (97.5%) were not considered related to intake of the study products (most common AEs were headache and nasopharyngitis). One AE was classified as definitely related to the Test product (headache) and one AE was classified as probably related to the Reference product (nausea).

## 4. Discussion

In this single-dose study conducted in healthy adults, an AA mixture formulated with Physiomimic Technology^TM^ significantly prolonged the release of AAs, lowering peak EAA levels in plasma (first co-primary endpoint), while maintaining an equivalent overall increase in plasma EAAs (second co-primary endpoint), compared to a reference amino acid mixture of similar composition but formulated without the novel coating technology. The aim of prolonging AA release and absorption is to avoid an AA C_max_ that exceeds anabolic capacity and promotes metabolic breakdown of AAs, while maintaining overall absorption for sustained protein synthesis. Avoiding excessively high AA C_max_ and prolonging absorption improves AA utilization for protein synthesis [10,22,23]. This improvement in nitrogen balance results from both increased protein synthesis and decreased metabolic breakdown of AAs [24]. A positive nitrogen balance after AA ingestion could be particularly important for PKU patients, and generally for all such patients with disorders of amino acid metabolism, where AA supplementation represents the major nitrogen source. To overcome the known limitations of immediate-release free AAs, several measures have been introduced in the dietary management of PKU patients, such as increasing the daily intake of phenylalanine-free protein substitute (i.e., at least 150% of normal dietary requirements) and administering multiple doses over the day. Nitrogen retention is improved when free AA mixtures are given in multiple doses over the day, rather than as a single bolus [25], and there is less fluctuation of blood phenylalanine concentrations. AA mixtures that mimic physiological absorption kinetics are expected to improve the rate of assimilation into protein and minimise oscillations of amino acid levels [17]. PT^TM^ could be one means to achieve this.

We chose EAAs for the primary endpoints because they are not synthesised endogenously and because they are often chronically deficient in the diet of patients with metabolic disorders such as PKU, making the patients strongly dependent on phenylalanine-free proteins or protein substitutes. The results obtained for the primary endpoints—lower peak levels of EAAs in plasma and sustained overall bioavailability of EAAs—were corroborated by similar results from the secondary analyses performed up to 7 h after product intake for LNAAs, BCAAs and total AAs. The secondary endpoints were designed as potentially confirmative observations to further characterise the effect of the engineered AA formulation on plasma AA profiles and to explore potential effects of AA absorption kinetics on selected metabolic parameters.

Formal comparisons between the Test product and MP or casein would not be informative, because they contain different proportions and relative quantities of AAs; however, secondary comparisons showed that the increase in plasma AAs with the Test product was prolonged also compared to MP and casein. It should be noted that the C_max_ of the food grade casein product employed was unexpectedly high, and not consistent with published reports [12,26,27] or with our previous experience in a porcine model, where a technical grade casein product and prototypes of the Test product had similar prolonged absorption kinetics [18].

The addition of extra tyrosine to AA mixtures used for dietary treatment of PKU is critical to compensate the lack of endogenous tyrosine production due to low or missing phenylalanine hydroxylase activity; however, plasma tyrosine concentrations can fluctuate greatly over the day in patients with PKU, and this may be exacerbated by supplementation with free AA mixtures [28,29,30]. Bioavailability of this conditionally essential AA was similar between the Test product and Reference product (AUC_420_ ratio 1.052; 90% CI 0.984, 1.125), whereas the Test product produced less variability.

Phenylalanine is not present in AA mixtures for dietary treatment of PKU, and their administration in patients with PKU has been associated with an unphysiological lowering of postprandial plasma phenylalanine levels [31], therefore, we assessed the effects of the Test product and the two immediate-release formulations (Reference and MP) on plasma phenylalanine levels. The two immediate-release formulations were associated with more pronounced fluctuations in plasma phenylalanine levels compared to the Test product. Postprandial plasma AA levels are influenced by several tightly controlled processes in addition to intestinal absorption (e.g., transportation, AA oxidation, protein degradation, anabolic processes). Plasma AA levels can influence several of these processes. When Giordano et al. infused increasing amounts of a balanced AA mixture in fasted healthy adults, the first effect observed was inhibition of protein catabolism, followed at higher AA doses by stimulation of protein synthesis and finally an increase in AA oxidation [32]. Our insulin data suggest that there is a stronger anabolic response after ingesting the immediate-release AA products. This could have stimulated more protein synthesis and uptake of AAs including phenylalanine from the blood; moreover, the observed decrease in plasma phenylalanine occurred between 45 and 90 min after ingestion, which corresponds to the timing of muscle protein synthesis reported in fasted healthy adults fed whey protein [33]. The effects of the Test product on plasma AA levels are likely to be mediated by several mechanisms. It will be important to determine its effect on AA profiles when co-administered with a carbohydrate source that stimulates a stronger insulin response. We conducted this study in healthy volunteers, therefore phenylalanine hydroxylase activity may have influenced plasma phenylalanine levels to a greater extent in our subjects than it would in patients with PKU.

Assessment of BUN provides an indication of the extent of oxidative metabolic breakdown of AAs. It is expected to increase when AAs are used as an energy source. The rise in BUN after ingesting the nitrogen sources in this study was significantly lower with the Test product compared to the Reference product. This finding is confirmed by the observation that less urea was excreted in urine after ingesting the Test product. Significantly lower BUN and urea excretion were observed also when the Test product was compared to MP, supporting a connection between prolonged absorption and reduced metabolic breakdown of AAs.

Ingestion of AAs stimulates insulin secretion [34] and we observed an insulin secretagogue effect with all four study products, followed by a reduction in blood glucose levels due to insulin-mediated glucose uptake by peripheral tissues. However, the Test product was associated with a lower insulin peak (at 30 min after intake) and less pronounced reduction in blood glucose levels (at 60 min) compared to the immediate-release AA products (Reference and MP). This is consistent with reports that ingesting intact protein results in a lower insulin peak at 30 min compared to ingesting free AA mixtures [35]. Several studies have shown that carbohydrate foods with a low glycaemic index induce higher satiety and lead to less voluntary food intake at a subsequent meal, compared to foods with a higher glycaemic index [36,37,38]. The less pronounced insulin and glucose excursions observed with the Test product may help to maintain a normal satiety response. The findings from this single-dose study may be predictive of sustained health benefits if a prolonged-release product is consumed regularly. The need for constant treatment over the life of the patient underscores the importance of developing AA mixtures that provide physiological absorption. This should play an important role in improving the management of patients with metabolic disorders like PKU.

The Test product was well tolerated, with 1 out of 30 subjects experiencing an adverse event (headache) that was seen as associated with product intake. The functional additives used in the PT^TM^ procedure are widely used by the food industry and are generally recognized as safe [39,40]. This point is important because patients with conditions like PKU must consume these products every day of their lives.

This study addresses dietary management with an AA mixture appropriate for PKU; however, the findings may be generalizable to other conditions that are managed with specific AA mixtures. These might include AA mixtures lacking methionine in homocystinuria [4], leucine, isoleucine and valine in maple syrup urine disease [5], phenylalanine and tyrosine in tyrosinemia [6], lysine and tryptophan in glutaric aciduria type 1 [7], and isoleucine and valine in some organic acidurias [8].

We have conducted this proof of concept study in healthy adults. Several studies suggest that important aspects of protein metabolism in patients with PKU are similar to those in the healthy population. Van Rijn et al. [41] reported no significant differences in postprandial insulin levels, protein turnover, or protein synthesis between healthy controls and patients with PKU. Thompson et al. [42] studied whole-body protein metabolism in patients with PKU and showed that protein turnover was comparable to that of healthy controls. Therefore, we expect that our results with a prolonged absorption AA formulation would be similar in patients with PKU.

## 5. Conclusions

This study shows that applying PT^TM^ to an AA mixture prolongs the increase in plasma AAs compared to free AAs. This is associated with modulation of several metabolic markers. It will be important to understand whether this can improve the long-term management of inborn errors of AA metabolism requiring low protein diets supplemented with specific AA mixtures. This information may be useful for those who would like to evaluate the Test product in clinical practice.

## Figures and Tables

**Table 1:** Peak blood EAA concentration (**C_max_**) and overall increase in plasma EAAs up to 300 min (AUC**_0-300min_**) after intake of Test and Reference products (co-primary endpoints).

**EAA C_max_ mmol/L, Mean (SD)**		**C_max_ Ratio**	***p*-Value ^‡^**
**Test**	**Reference**		
1.77 (0.253)	2.44 (0.368)	0.726	<0.0001
**EAA AUC_0-300min_ (mmol/L) min, Mean (SD)**		**AUC_0-300min_ Ratio**	**90% CI**
**Test**	**Reference**		
396 (45.0)	444 (46.2)	0.890	0.865, 0.915 *

* Value within the predefined bioequivalence range. ^‡^ Linear mixed effect model EAA, essential amino acids; SD, standard deviation; AUC, area under the time-concentration curve; CI, confidence interval.

**Table 2:** The peak plasma amino acid concentrations (C_max_) after ingesting the Test or Reference products (secondary endpoints).

AA Group	C_max_ mmol/L, Mean (SD)		C_max_ Ratio	*p*-Value ^‡^
	Test	Reference		
LNAAs	1.27 (0.175)	1.87 (0.301)	0.677	<0.0001
BCAAs	0.693 (0.106)	1.20 (0.221)	0.579	<0.0001
Total AAs	3.57 (0.468)	4.59 (0.576)	0.775	<0.0001

^‡^ Linear mixed effect model AA, amino acid; LNAA, large neutral amino acid; BCAA, branched-chain amino acid; SD, standard deviation.

**Table 3:** Overall increase in plasma levels of the indicated amino acids up to 7 h after ingesting the Test or Reference products (secondary endpoints).

AA Group	AUC_0–420min_ (mmol/L) *min, Mean (SD)		AUC_0–420min_ Ratio	90% CI
	Test	Reference		
EAAs	509 (57.1)	549 (55.1)	0.924	0.900, 0.950 *
LNAAs	385 (43.2)	424 (43.9)	0.908	0.885, 0.932 *
BCAAs	206 (28.3)	240 (29.6)	0.860	0.836, 0.885 *
Total AAs	1106 (129)	1176 (97.2)	0.937	0.909, 0.966 *

* value within the predefined bioequivalence range. AA, amino acid; EAA; essential amino acid; LNAA, large neutral amino acid; BCAA, branched-chain amino acid; SD, standard deviation; AUC, area under the time-concentration curve; CI, confidence interval.

**Table 4:** Plasma amino acid concentrations 7 h after administration (C_last_).

AA Group	C_last_ mmol/L, Mean (SD)		C_last_ Ratio	*p*-Value ^‡^
	Test	Reference		
EAAs	0.822 (0.119)	0.780 (0.0882)	1.054	0.0158
LNAAs	0.628 (0.0920)	0.595 (0.0677)	1.055	0.0123
BCAAs	0.316 (0.0596)	0.281 (0.0525)	1.126	0.0001
Total AAs	2.09 (0.275)	2.06 (0.207)	1.012	0.5658

^‡^ Statistical test RM-ANCOVA. AA, amino acid; EAA; essential amino acid; LNAA, large neutral amino acid; BCAA, branched-chain amino acid; SD, standard deviation.

**Table 5:** Peak plasma AA concentrations after ingesting the Test product or the indicated secondary comparators.

AA Group	C_max_ mmol/L, Mean (SD)			Comparisons			
				Test vs. MP		Test vs. Casein	
	Test	MP	Casein	C_max_ Ratio	*p*-Value ^‡^	C_max_ Ratio	*p*-Value ^‡^
EAAs	1.77 (0.253)	2.12 (0.314)	2.18 (0.379)	0.835	<0.0001	0.818	<0.0001
LNAAs	1.27 (0.175)	1.64 (0.226)	1.68 (0.282)	0.775	<0.0001	0.759	<0.0001
BCAAs	0.693 (0.106)	1.043 (0.149)	1.03 (0.189)	0.664	<0.0001	0.676	<0.0001
Total AAs	3.57 (0.468)	4.11 (0.585)	4.16 (0.613)	0.870	<0.0001	0.860	<0.0001

^‡^ Linear mixed effect model AA, amino acid; EAA; essential amino acid; LNAA, large neutral amino acid; BCAA, branched-chain amino acid; SD, standard deviation; C_max_, maximum plasma concentration; MP, marketed product.

**Table 6:** Overall increase in plasma AA levels over 7 h after ingesting the Test product or the indicated secondary comparators.

AA Group	AUC_0–420min_ (mmol/L) *min, Mean (SD)			Comparisons			
				Test vs. MP		Test vs. Casein	
	Test	MP	Casein	AUC_0–420min_ Ratio	90% CI	AUC_0–420min_ Ratio	90% CI
EAAs	509 (57.2)	556 (54.6)	543 (55.4)	0.917	0.892, 0.942 *	0.936	0.910, 0.961 *
LNAAs	385 (43.2)	435 (39.0)	422 (40.3)	0.887	0.864, 0.910 *	0.911	0.888, 0.935 *
BCAAs	207 (28.3)	263 (26.1)	248 (27.3)	0.758	0.763, 0.807	0.829	0.806, 0.852 *
Total AAs	1106 (129)	1170 (123)	1160 (122)	0.945	0.917, 0.974 *	0.952	0.923, 0.981 *

* Value within the predefined bioequivalence range. AA, amino acid; EAA; essential amino acid; LNAA, large neutral amino acid; BCAA, branched-chain amino acid; SD, standard deviation; AUC, area under the concentration-time curve; MP, marketed product; CI, confidence interval.

**Table 7:** Total urea excreted in urine in the first 5 h after ingesting study products.

Statistics	Test	Reference	Casein	MP
LSM, mmol 95% CI	40.1 36.0, 44.2	45.6 41.6, 49.6	43.7 39.7, 47.7	39.9 35.8, 43.9
**Comparisons with Test**	**LSM Difference**	**95% CI**	***p*-Value**	
vs Reference	−5.52	−9.52, −1.52	0.0074 *	
vs Casein	−3.62	−7.61, 0.37	0.075	
vs MP	0.203	−3.85, 4.25	0.92	

* statistically significant (RM-ANOVA) CI, confidence interval; LSM, least square mean; MP, marketed product.

**Table 8:** Plasma levels of metabolic markers over the first 5 h following ingestion of the indicated products.

Parameter	AUC_0-300min_ Mean (SD)				Ratio of Geometric LSM (90% CI)		
	Test	Reference	MP	Casein	Test vs. Reference	Test vs. MP	Test vs. Casein
BUN, (mmol/L) *min	1357 (201)	1573 (266)	1423 (241)	1476 (292)	0.868 (0.837, 0.900)	0.957 (0.923, 0.993)	0.927 (0.894, 0.962)
Insulin, (U/L) *min	2.14 (1.01)	2.70 (1.38)	2.59 (1.08)	2.60 (1.34)	0.785 (0.716, 0.861)	0.803 (0.732, 0.880)	0.819 (0.748, 0.898)
Glucose, (mmol/L) *min	1621 (91)	1609 (93)	1610 (86)	1600 (111)	1.005 (0.992, 1.019)	1.006 (0.992, 1.019)	1.012 (0.999, 1.026)

AUC, area under the concentration-time curve; LSM, least squares mean; BUN, blood urea nitrogen; SD, standard deviation; MP, marketed product.

## Supplementary Materials

The following are available online at https://www.mdpi.com/2072-6643/12/6/1653/s1, Figure S1: Schematic representation of immediate release vs. prolonged-release absorption kinetics, Figure S2: Study design showing the randomization sequence applied. Table S1: Complete enrolment criteria, Table S2: Composition of the Test and Reference products used in the study.
