# Supplementary material for: Amino Acid Plasma Profiles from a Prolonged-Release Protein Substitute for Phenylketonuria: A Randomized, Single-Dose, Four-Way Crossover Trial in Healthy Volunteers
Source: Nutrients. 2020 Jun 2;12(6):1653. doi: 10.3390/nu12061653 (PMC7352445; doi:10.3390/nu12061653)
Supplement: Supplementary file 1 [file nutrients-12-01653-s001.zip › Suppl figures nd Tables/Supplemental Table S1. Inclusion and Exclusion criteria.docx]

**Inclusion criteria**

To be eligible for the trial, the subjects needed to fulfil the following inclusion criteria:

- Males and females aged 18-45 years (limits included);
- Weight (kg) within the range of 55-85 kg and body mass index (BMI) ≤ 30 kg/m^2^;
- Willing and able to understand and sign the written informed consent form;
- Willing to consume medical nutrition products, specifically AA preparations (L-AAs/protein substitutes), and to follow the dietary scheme as required by the protocol;
- Good general health status, as documented by normal findings in the medical history, physical examination, 12-lead ECG, vital signs (body temperature, systolic and diastolic blood pressure, heart rate after a 3-min rest), and laboratory parameters*. Values for the laboratory parameters were compared with normal ranges from the laboratory. Parameters out of these normal ranges were carefully evaluated by the Investigator who then decided whether to consider them “clinically not relevant” or “clinically relevant” for the current study.
- Non-smokers or not current smokers.

* Screening laboratory tests included:

- haematological and biochemical tests: blood count (total and differential white blood cells; red blood cells; haemoglobin, haematocrit, thrombocytes); C-reactive protein (CRP); insulin and glucose, liver and kidney function tests (aspartate aminotransferase, alanine aminotransferase, γ-glutamyl transferase, bilirubin, alkaline phosphatase, creatinine); electrolytes (Na^+^, K^+^); serum proteins (albumin, prealbumin), creatine kinase, prothrombin time; total cholesterol, triglycerides;

- blood pregnancy test: human chorionic gonadotropin (hCG) for females of childbearing potential;

- virological tests: HIV, Hepatitis B and C.

**Exclusion criteria**

The presence of any of the below listed exclusion criteria excluded the subjects from the inclusion in the trial:

- Pregnancy, lactation or planned pregnancy*;
- History of clinically significant diseases or malfunctions including, but not limited to, gastrointestinal, renal, hepatic, pulmonary, cardiovascular or endocrine disease, haemophilia, hyperlipidaemia, impaired glucose tolerance or diabetes (type 1 or 2), or anaemia;
- Current illnesses that could interfere with the study [for instance: severe inflammation (elevated CRP and/or haematocrit), prolonged diarrhoea, regurgitation];
- Use of any medication that might significantly affect protein synthesis or turnover (like anabolic steroids, growth hormone, nonsteroidal anti-inflammatory drugs, hormone replacement therapy, etc.), upon decision of the Investigator;
- Clinically significant abnormalities in screening laboratory values (especially blood count, liver and kidney function tests, blood glucose, serum albumin):
- Any medical condition deemed to be exclusionary by the Investigator (reasoning needed to be provided);
- Any current participation in another clinical trial involving investigational or marketed products in the 3 months prior to the inclusion in this clinical trial;
- Blood donation of ≥250 mL within the past 3 months;
- Vegetarian diet (i.e. vegan, lacto-vegetarian, lacto-ovo vegetarian, pesco-vegetarian) or any food allergy towards any of the ingredients in the study products (like allergies to cow’s milk or to products derived from milk) or to ingredients of snack bars/meals to be administered during the study;
- Abnormal diets or substantial changes in eating habits with the past 4 weeks;
- Positive result in HIV test, or Hepatitis B or C tests;
- Unwilling to provide a written informed consent;
- Likelihood that the subject will not be compliant or not cooperative during the study, in the judgement of the Investigator.

* If a female was of childbearing potential, verification on contraception use was needed at screening, in addition to the evaluation of hCG in blood; finally, pregnancy tests were performed at each test day, before study product’s intake (urine stick test).

The use of a reliable contraceptive method - such as the pill or a combination of mechanical methods (IUD or diaphragm together with condom) in case of sexual intercourse - was mandatory for females of childbearing potential. For males, in case of sexual intercourse, the use of reliable contraceptive method by them and their mates was mandatory in order to avoid pregnancy in their mates.

Other restrictions and requests:

- subjects were not allowed to use any AA nor protein enrichment (protein powders or AA supplements) starting from 2 weeks before the randomization and during the whole study;
- use of genetically modified probiotics or LNAAs was not allowed starting from 2 weeks before the randomization and during the whole study;
- no alcoholic beverage was allowed during the 24 hours prior to each test day;
- no strenuous physical activity was to be performed during the week before each study product intake;
- subjects had to follow a standardized diet (with a moderate protein content) in the two days preceding each test day;
- use of caffeine-containing products was not allowed during the test days.
