# Supplementary material for: Amino Acid Plasma Profiles from a Prolonged-Release Protein Substitute for Phenylketonuria: A Randomized, Single-Dose, Four-Way Crossover Trial in Healthy Volunteers
Source: Nutrients. 2020 Jun 2;12(6):1653. doi: 10.3390/nu12061653 (PMC7352445; doi:10.3390/nu12061653)
Supplement: Supplementary file 1 [file nutrients-12-01653-s001.zip › Suppl figures nd Tables/Supplemental Table S2.AA composition TEST-REFERENCE.pdf]

Specifications for the Test product
(APR-1301-01 modified-release AA mixture)

Ingredient	g in a sachet of 24g	g for 100g of product
L-Serine	0.6100	2.5417
L-Threonine	0.9000	3.7500
L-Leucine	2.0700	8.6250
Glicine	0.9000	3.7500
L-Alanine	0.5400	2.2500
L-Arginine	0.7200	3.0000
L-Cystine	0.3600	1.5000
L-Glutamine	3.6000	15.0000
L-Histidine	0.5032	2.0967
L-Aspartic Acid	1.0800	4.5000
L-Proline	1.0800	4.5000
L-Isoleucine	0.9900	4.1250
L-Lysine	1.2600	5.2500
L-Tryptophan	0.3600	1.5000
L-Valine	0.9000	3.7500
L-Methionine	0.2500	1.0417
Taurine	0.0500	0.2083
L-Carnitine	0.0200	0.0833
L-Tyrosine	1.8000	7.5000
**Ingredient**	**mg in a sachet of 24g**	**mg for 100g of product**
choline	77.143	321.429
inositol	51.429	214.288
vitamin A	0.311	1.296
vitamin D	0.006	0.025
vitamin E	3.231	13.463
vitamin K	0.024	0.100
vitamin C	32.306	134.608
thiamin	0.471	1.963
riboflavin	0.454	1.892
niacin	6.429	26.788
vitamin B6	0.621	2.588
folic acid	0.064	0.267
vitamin B12	0.001	0.0042
biotin	0.013	0.054
pantothenic acid	2.571	10.713
potassium	300.017	1250.071
calcium	321.429	1339.288
magnesium	72.857	303.571
iron	5.571	23.213
zinc	3.429	14.288
copper	0.343	1.429
manganese	0.600	2.500
selenium	0.014	0.058
chromium	0.011	0.046
molybdenum	0.021	0.088
iodine	0.054	0.225
phosphorus	249.694	1040.392
chlorine	0.1791	0.7462
sodium	5.934	24.724

Specification for the REFERENCE product (APR-1301-01 immediate-release AA mixture)

Ingredient	g in a sachet of 24g	g for 100g of product
L-Serine	0.6100	2.5417
L-Threonine	0.9000	3.7500
L-Leucine	2.0700	8.6250
Glicine	0.9000	3.7500
L-Alanine	0.5400	2.2500
L-Arginine	0.7200	3.0000
L-Cystine	0.3600	1.5000
L-Glutamine	3.6000	15.0000
L-Histidine	0.5032	2.0967
L-Aspartic Acid	1.0800	4.5000
L-Proline	1.0800	4.5000
L-Isoleucine	0.9900	4.1250
L-Lysine	1.2600	5.2500
L-Tryptophan	0.3600	1.5000
L-Valine	0.9000	3.7500
L-Methionine	0.2500	1.0417
Taurine	0.0500	0.2083
L-Carnitine	0.0200	0.0833
L-Tyrosine	1.8000	7.5000
**Ingredient**	**mg in a sachet of 24g**	**mg for 100g of product**
choline	77.143	321.429
inositol	51.429	214.288
vitamin A	0.311	1.296
vitamin D	0.006	0.025
vitamin E	3.231	13.463
vitamin K	0.024	0.100
vitamin C	32.306	134.608
thiamin	0.471	1.963
riboflavin	0.454	1.892
niacin	6.429	26.788
vitamin B6	0.621	2.588
folic acid	0.064	0.267
vitamin B12	0.001	0.0042
biotin	0.013	0.054
pantothenic acid	2.571	10.713
potassium	300.017	1250.071
calcium	321.429	1339.288
magnesium	72.857	303.571
iron	5.571	23.213
zinc	3.429	14.288
copper	0.343	1.429
manganese	0.600	2.500
selenium	0.014	0.058
chromium	0.011	0.046
molybdenum	0.021	0.088
iodine	0.054	0.225
phosphorus	249.694	1040.392
chlorine	0.1791	0.7462
sodium	5.934	24.724
