# Supplementary figures and images for: Amino Acid Plasma Profiles from a Prolonged-Release Protein Substitute for Phenylketonuria: A Randomized, Single-Dose, Four-Way Crossover Trial in Healthy Volunteers
Source: Nutrients. 2020 Jun 2;12(6):1653. doi: 10.3390/nu12061653 (PMC7352445; doi:10.3390/nu12061653)

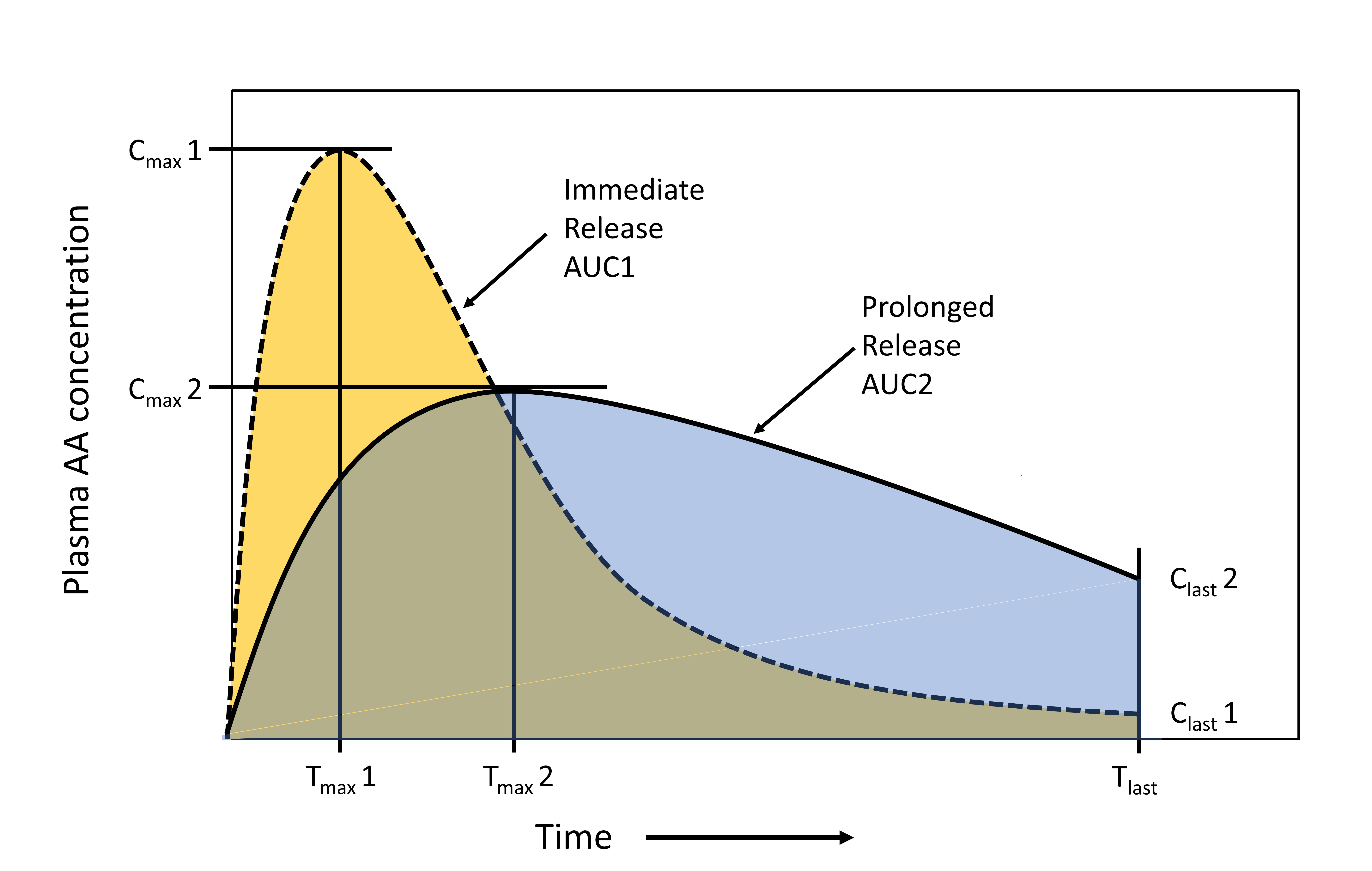

Supplement: Supplementary file 1 [file nutrients-12-01653-s001.zip › Suppl figures nd Tables/Supplemental figure 1.jpg]

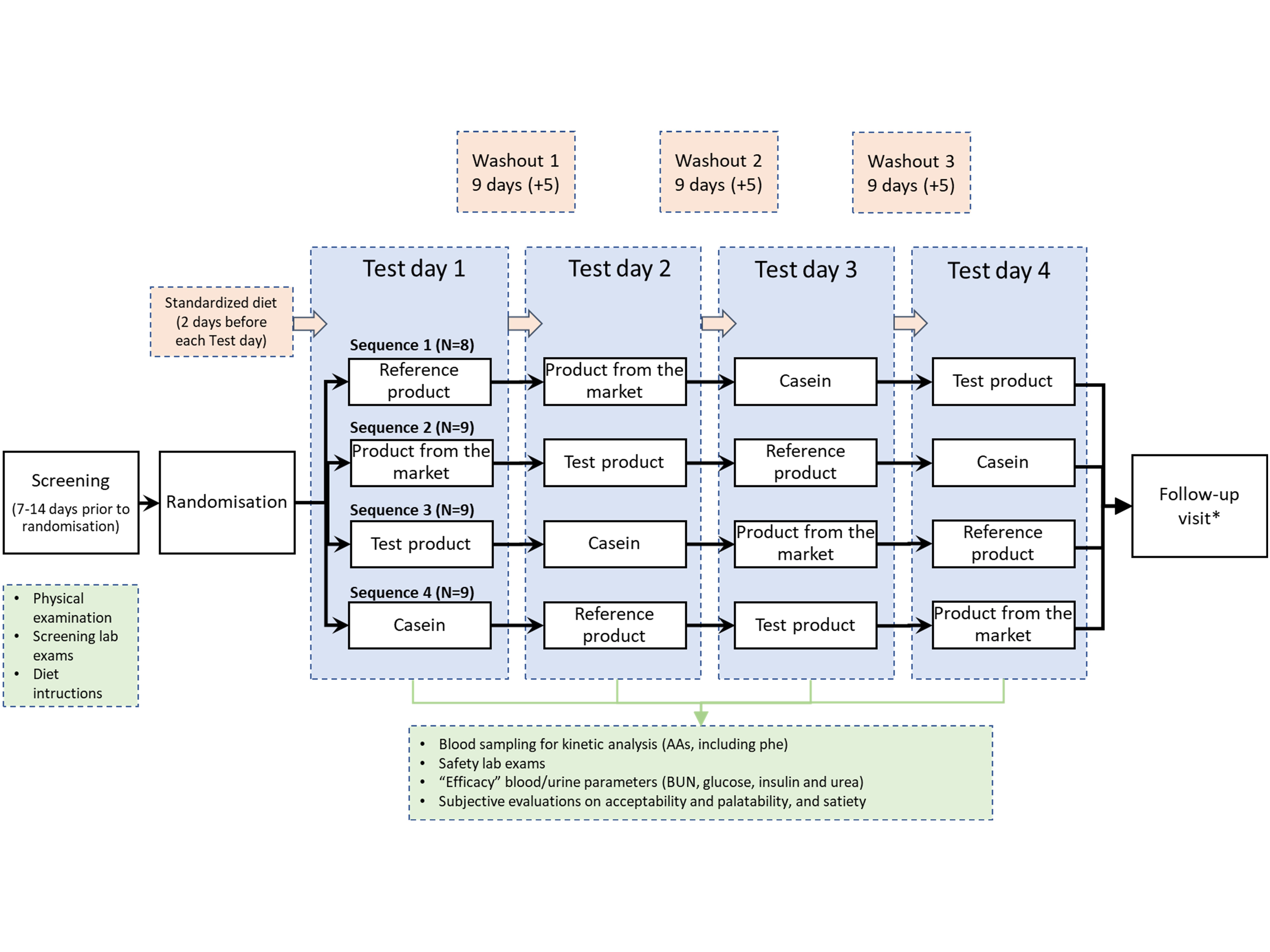

Supplement: Supplementary file 1 [file nutrients-12-01653-s001.zip › Suppl figures nd Tables/Supplemental figure 2.jpg]
